# In Vitro Propagation of *Origanum scabrum* (Boiss. & Heldr.): An Endemic Medicinal Plant of Greece

**DOI:** 10.3390/plants12112118

**Published:** 2023-05-26

**Authors:** Alexios A. Alexopoulos, Epaminondas Kartsonas, Stavros Karras, Eleni Mavrommati, Spyridon A. Petropoulos, Maria Papafotiou

**Affiliations:** 1 Laboratory of Agronomy, Department of Agriculture, University of the Peloponnese, Antikalamos, 241 00 Kalamata, Greece; s.karras@uop.gr (S.K.); e.mavrommati@uop.gr (E.M.); 2 Laboratory of Floriculture, Department of Agriculture, University of the Peloponnese, Antikalamos, 241 00 Kalamata, Greece; 3 Laboratory of Vegetable Production, Department of Agriculture, Crop Production and Rural Environment, University of Thessaly, Fytokou Street, 384 46 Volos, Greece; spetropoulos@uth.gr; 4 Laboratory of Floriculture and Landscape Architecture, Faculty of Crop Science, Agricultural University of Athens, Iera Odos 75, 118 55 Athens, Greece; mpapaf@aua.gr

**Keywords:** medicinal and aromatic plant, explant collection date, ex vitro establishment, Lamiaceae, micropropagation, node position

## Abstract

The aim of the study was to develop an efficient micropropagation protocol of *Origanum scabrum*, which will allow its commercial exploitation in the pharmaceutical and horticultural industries. First, the effect of the date of the explant collection (20 April, 20 May, 20 June, 20 July, 20 August) and the position of the explant on the plant stem (shoot apex, 1st node, 3rd node, 5th node) on the establishment of in vitro cultures was studied (1st experiment: Stage I). Next, the effect of temperature (15 °C, 25 °C) and the node position (microshoot apex, 1st node, 5th node) on the microplant production and ex vitro survival of plantlets was studied (2nd experiment: Stage II). The optimum season to collect explants from wild plants was shown to be during the vegetative growth of the plants (April to May), while the shoot apex and the 1st node were the most suitable explants. For the proliferation and production of rooted microplants, the best results were obtained from single-node explants excised from microshoots produced from 1st node-explants collected on 20th of May. Temperature did not affect microshoot number, leaf number and the percentage of rooted microplants, while microshoot length was higher at 25 °C. Moreover, microshoot length and the percentage of rooted microplants were higher in those derived from apex explants, while the survival of plantlets was not affected by treatments and ranged between 67% and 100%.

## 1. Introduction

**Figure 1:**
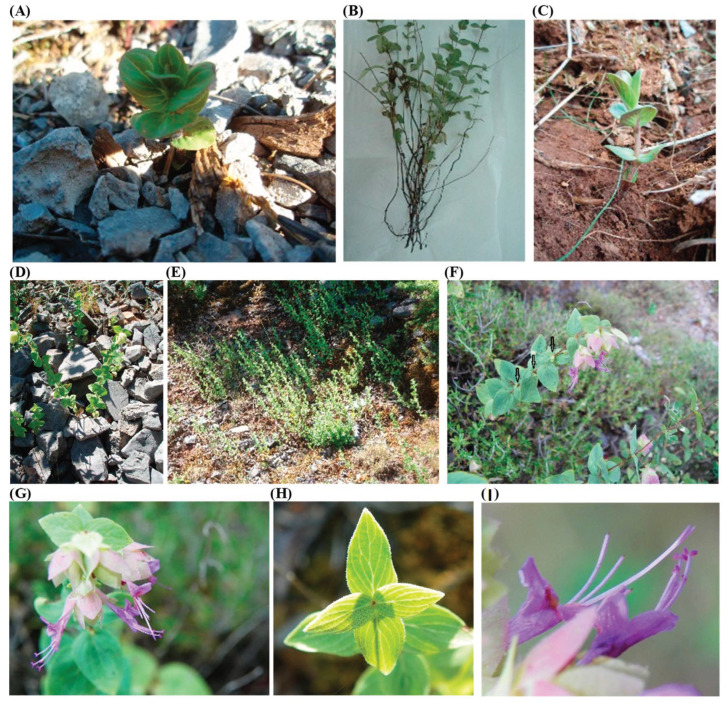
*Origanum scabrum* plant: (**A**) Early growth of a wild plant in March, (**B**) Naturally dried aerial plant parts in August, (**C**) Emergence of new vegetation from a rhizome, (**D**,**E**) Wild plants with erect stems and no lateral shoots, (**F**) Wild plant with lateral shoots near the top of the stem, (**F**,**G**) Inflorescences, (**H**) Leaves, (**I**) Flower.

*Origanum scabrum* (Boiss. & Heldr.) belongs to the Lamiaceae family and it is a rare endemic plant species of Greece; it is native to Mount Taygetos and Mount Parnonas in the Southern Peloponnese as well as to Mount Dirfi in Evia island [1,2,3]. It is a perennial, herbaceous wild plant with thin shoots (Figure 1A), while the aerial part of the plant dries up during summer (Figure 1B). Every year (usually during March) new stems are grown from the buds of the underground shoots—rhizomes (Figure 1C). The above-ground stems are erect (Figure 1D,E) growing to a height of 10–30 cm, although in some favorable environmental conditions they may exceed 40 cm [4]. Each node of the stem bears two opposite leaves and two axillary buds which do not always produce lateral shoots (Figure 1D,E). The leaves are simple, pointed, cordate, and smooth with characteristic stiff hairs on the periphery (Figure 1F,H), while the flowers which appear in a terminal inflorescence (Figure 1F) are small, with petals of lilac-pink color and pink—purple bract leaves at the base of each flower (Figure 1D,G).

The aerial part of the plant is rich in phenolic compounds, has a strong aroma and contains essential oils in a concentration ranging from 0.6% to 4%, depending on the collection site and date [5,6], as observed in other wild endemic Lamiaceae species in the Mediterranean Basin [7]. The essential oil consists of 28 compounds (they make up 98% of the essential oil), with carvacrol being the major compound detected in concentrations ranging from 66.7% [6] to 74.9% [5]. According to Demetzos et al. [6], other compounds detected in relatively high concentrations include p-cymene (7.8%), y-terpinene (3.6%), caryophyllene oxide (2.1%) and manool (2.7%). The essential oils of the plant possess strong antimicrobial activity against *Staphylococcus aureus*, *S. epidermidis*, *Enterobacter cloacae*, *Klebsiella pneumoniae*, *Pseudomonas aeruginose*, *Candida albicans*, *C. tropicalis* [5], which could be attributed to the high contents of carvacrol [8]. The wild plants are widely consumed by local people (selected or/and traded in local folk markets) and they are highly appreciated for their medicinal properties (e.g., used as decoctions against stomachache) and their spicy flavor (e.g., used in cooking and in pastry making) [9,10,11]. Additionally, the growth habit of stems and the appealing color of leaves and inflorescences, make it possible to use the species for ornamental purposes (e.g., in rock gardens, green roofs, etc.).

The plant has a limited distribution (grows in high altitudes of 900–1800 m) and is mainly found in rocky areas or in forest gaps, e.g., in forests of *Abies cephalonica* (Loudon) and *Pinus nigra* subsp. *pallasiana* [(Lamb.) Holmboe] [3]. According to Tan and Iatrou [3], other plant species grown in *O. scabrum* natural habitats are *Berberis cretica* (L.), *Daphne oleoides* (Schreb.), *Juniperus oxycedrus* (L.) and *Juniperus foetidissima* (Willd.) Although it is not referred in the IUCN list, factors such as its limited distribution and anthropogenic activities related to road constructions and overgrazing can lead to significant limitation of plant populations and increase the risk of genetic erosion. In addition, the irrational collection of native plants for human consumption may put the species under threat [12], as it has been reported for several medicinal plants native in the Mediterranean region [10,11,13]. These reasons may indicate the need to consider the addition of the plant species in the IUNC list. Therefore, the production of propagating material in a large scale not only allows the cultivation of endemic plant species and contributes to their sustainable commercial exploitation, but also decreases the risks of genetic erosion and the extinction of valuable genetic resources [10,13]. Similarly to other species of the genus *Origanum*, there are several obstacles in the production of propagating material mainly due to the limited rooting of the cuttings and the sterility or the low germination rate of the seeds [13,14]. Under these circumstances, micropropagation technique enables the production of healthy plants within a short period of time and in large quantities, regardless of the environmental conditions and with no requirements for wild plants collection, while at the same time it facilitates the effective conservation of genetic material [11,15,16,17].

So far, different plant parts such as seeds, young seedlings, mature plants, cotyledons, hypocotyl and root segments for rapid multiplication through callus have been used as explants for the establishment of in vitro cultures of *Origanum* species [18,19]. Moreover, in many species of the same genus micropropagation is achieved using the shoot apex (terminal bud-apical meristem) [14,20,21,22] or axillary buds [23,24,25].

To the best our knowledge, there is scarce literature concerning the in vitro propagation of *O. scabrum*. Therefore, the aim of the study was to develop a simple and efficient protocol for the commercial micropropagation of the species, which will allow its cultivation and commercial exploitation, as well as the selection of superior genotypes and the conservation of genetic material.

## 2. Results and Discussion

### 2.1. The Effect of Node Position and Collection Date of Explants on the Establishment of In Vitro Cultures

Surface sterilization of explants was successful (92–100%), and the success rate was not affected by either the date of explant collection or the node position (node order on the stem). Such a high percentage of uninfected explants has been also achieved in *O. vulgare* ssp. *hirtum* [(Link) A.Terracc.] by immersing axillary buds in 20% commercial sodium hypochlorite solution (0.98% *w*/*v*) for 20 min [24]. Therefore, it is not suggested to use ethyl alcohol (50–70% *v*/*v*) or higher concentrations of sodium hypochlorite (1–2% *w*/*v*), as it has been reported in other *Origanum* species [26]. Regardless of solution composition, slight mechanical stirring of surface sterilization solution is recommended paying special care to not injuring the explants [27,28].

Both factors (node position and explant collection date), as well as their interaction, affected significantly the percentage of the explant response in the medium without the addition of plant growth regulators (Table 1). Twenty-five days after the establishment of in vitro cultures, apical shoot explants responded at higher rates (80–93.3%) when collected on 20 April–20 June. Similarly, 1st node explants collected on 20 April and 20 May responded at higher rates (90–96.7%) than those of the later dates. Moreover, the response of the 3rd node (23.3–56.7%) and the 5th node (6.7–23.3%) explants was significantly higher in the collections of 20 April compared to later collection dates. It is worth noting that explant response percentage was very low on 20 August (0–16.7%), regardless of their position on the shoot. Explants from shoot apex and/or the 1st node recorded the highest response percentage for all the tested collection dates.

The time of explant collection is well known to influence explant response in in vitro cultures of many plant species [29]. For example, the seasonal variation of bud response in nodal explants has been observed in trees, e.g., *Terminalia bellirica* [(Gaertn.) Roxb.] [30] and *Tecomella undulata* [(Sm.) Seem.] [31]. Moreover, Mederos and Enrquez [32] observed a higher response of single node explants (1 cm stem with one bud) in *Rosa hybrida* (L.) when collected from young shoots (length of about 10 cm and six axillary buds), compared to those obtained from flowering shoots. In addition, Bressan et al. [33] reported that the node position significantly affected the ability of axillary buds to produce microshoots in *Rosa hybrida*.

Apart from collection time, conditions of culture may also affect explant response after the establishment of in vitro cultures. Long photoperiod seemed to be suitable for high response of *O. scabrum* explants (although short days were not tested), in agreement with other studies of *Origanum* species, e.g., *O. compactum* (Benth.) [22], *O. sipyleum* (L.) [34], and *O. acutidens* [(Hand.-Mazz.) Ietsw.] [35]. On the other hand, in other studies on in vitro cultures of *Origanum* spp. temperature ranged at higher levels, e.g., 22 °C in *O. vulgare* × *applii* [14] and 26 °C in *O. syriacum* (L.) and *O. ehrenbergii* (Boiss.) [13] than those in our study (daily variation between 12 °C during dark and 18 °C during light period). It has been reported, however, that temperatures close to those prevailing in areas where a plant is native are more suitable for the establishment of in vitro cultures, especially in the case of endemic species [36]. Temperatures chosen for the establishment of *O. scabrum* explants in our study were close to temperatures that prevail in the area of Dyrrachion, where the average temperature of 16–18 °C is usually recorded between April and August. On the other hand, establishing in vitro cultures at temperatures much lower or much higher than those of the area where mother plants grow, favors the exposure of explants to stressful conditions which may result in the production of high concentrations of phenolic substances, as shown in *Lavandula viridis* (L’Hér.) and *Thymus lotocephalus* (G. López & R. Morales) [37]. It is already confirmed that the oxidation of phenolic substances may lead to the formation of dark compounds (browning of tissues) and further result in the death of explants or young microplants, especially if explants derived from mother plants grown in the field compared to explants derived from microplants [38].

**Figure 2:**
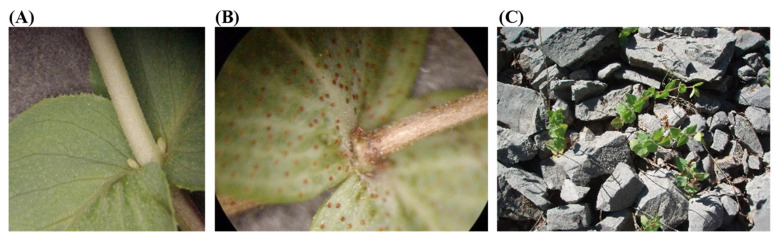
(**A**) Axillary buds on shoots collected on 20 May (vegetative phase of the plant), (**B**) Axillary buds in senescent shoots selected on 20 July. (**C**) Wild plants with no lateral shoots on 20 May.

The number of microshoots per explant was significantly affected by the collection date and the position of the explant (node order) οn the shoot of the mother plant (Table 2). In particular, apical shoot explants produced one microshoot when collected on 20 April, 20 June and 20 July, while only half of the explants produced microshoots in the collection of 20 August. Additionally, the apical shoot explants collected on 20 June and 20 July formed inflorescent (Figure 2D) at a rate of 40 and 67%, respectively. The same response has been observed in other species, such as *Salvia africana-lutea* (L.) [39], while the transition from vegetative to reproduction phase limits the further longitudinal growth of microshoots and the production of new nodes.

On the other hand, explants obtained from the 1st node produced the highest number of microshoots (1.5–1.6) than the rest of node positions in collection dates between 20 April and 20 June, due to the existence of two buds per node (Figure 2A), as it has been already reported for *Origanum vulgare* (L.) [40]. Similarly, the number of microshoots produced in explants obtained from the 3rd and 5th node was the lowest regardless of collection date, while no microshoots were recorded when explants were collected between 20 June (5th node explants) and 20 August (1st and 3rd node; no response was recorded for 5th node explants).

The reduced capacity of lower-node explants (i.e., 3rd and 5th node) to produce microplants, especially when the plants are in the vegetative phase (e.g., collection on 20 April and 20 May), is likely to be linked with the effect of endogenous hormones [41]. It has been observed that native *O. scabrum* plants do not always form lateral shoots when they grow in their natural environment (Figure 2C), and if they do this usually occurs in buds located in the upper nodes of the shoot (Figure 1F). However, the inability of axillary buds to produce lateral shoots with increasing shoot age (e.g., collection of 1st node on 20 June and 20 July) may also be related to the effect of the shoot physiological senescence which results in the inactivation of the meristems of the axillary buds (Figure 2B). Similarly, nodal explants of *Rosa setigera* (Michx.) [42] and *Ginkgo biloba* (L.) [43] collected at the vegetative phase of mother plant had a higher response to tissue culture than those collected from mature plants. Thus, these differences between nodes of different position οn the shoot could be due to the shoot senescence starting from the basal part, as in *Scutellaria* spp. [44], the levels of endogenous hormones (e.g., ABA, ethylene), and the toxic effects of phenolic compounds or secondary metabolites etc. [41,45].

Therefore, the balance of endogenous hormones seems to be an important factor for the response of explants to micropropagation techniques. According to the literature, the addition of cytokinins and/or auxins in the medium promotes the production of microshoots in single or multiple node explants of *Origanum* species, such as *O. bastetanum* (Socorro, Arrebola & Espinar) [46], *O. minutiflorum* (O. Schwarz & P.H. Davis) [47] and *O. acutidens* [35] by overcoming the apical dominance. However, Goleniowski et al. [14] reported the addition of BA and NAA in the media did not affect the number of nodes produced per single node explant and the multiplication efficiency of ‘Mendocino’ oregano (*O. vulgare* × *applii*). On the other hand, cytokinins may have inhibitory effect on the development of lateral buds in *Rosa hybrida*, while relatively high cytokinins concentration may lead to difficulties in microplant rooting [33]. According to Kumar and Bhardwaj [26], the use of cytokinins in growing media for in vitro cultures of *Origanum* species can lead to callus formation and the production of not only axillary shoots but also of adventitious shoots, increasing the risk of regenerating plants with different genetic identity and, additionally, limiting microshoot length which may lead to difficulties in cutting nodes from microshoots for further subcultures [48]. In our study, all explants produced axillary shoots but no adventitious shoots, a finding that has been also observed in *Majorana hortensis* (Moench) [49]. Thus the two stage method followed in this work: (a) the establishment of in vitro culture with single node explants (from shoot apex and axillary buds) to produce unbranched shoots (Stage I); (b) collection of single node explants from shoots of stage I for the propagation (proliferation) of microshoots and the production of rooted microplants (Stage II), seems to be simpler and it can be achieved without the use of plant growth regulators for shoot proliferation or even rooting. This method, although it usually has a lower multiplication rate, is popular for commercial micropropagation and allows maintaining the genetic purity of selected clones [48,50,51,52].

Forty-five days after establishment of the in vitro cultures, the mean number of nodes (reproductive units) was higher in microplants from apical shoot explants (1.58–2.67) and 1st node explants (1.17–2.60) compared to those from the 3rd (0–0.97) and 5th (0–0.50) node explants on all collection dates, except 20 August (Table 3). Moreover, collection of explants between 20 April and 20 June resulted in higher number of nodes per microplant in the case of apical shoot explants, while for explants obtained from lateral nodes of an early collection, 20 April and 20 May, gave the best results. This parameter is pivotal for the successful micropropagation of the species since it allows the shorter duration of in vitro culture until obtaining single node explants for subculture and further production of a suitable microplant. Moreover, the production of higher number of microshoots per explant observed in the 1st node (1.5–1.6 per explant, Table 2) in the collections of 20 April and 20 May and consequently the availability of 1.5–1.6 new microplants, along with the high number of nodes (2.6, Table 3) for the same collection dates, point out 1st nodes as the preferred explant for the establishment of in vitro cultures of *O. scabrum* using the two-stage protocol of our study.

### 2.2. Effect of Temperature and Position of Single Node Explants on the Production of Microplants

The number of microshoots produced per single node explant, 35 days after the onset of subculture, was not significantly affected by temperature during culture, except when explants of the 5th node were used where microshoot number was reduced at the higher temperature (25 °C) (Table 4). Moreover, the number of microshoots was higher in microplants produced by explants of the 1st and 5th node than the shoot apex at both temperatures, except for microplants of 35 days and 25 °C. This finding suggests that nodes from the lower part of microshoots (e.g., the 5th node) forms buds which can produce similar number of microshoots as those produced from nodes near the shoot apex (e.g., the 1st node), in contrast to what we observed in explants taken from wild plants where the highest number of microshoots was recorded for the 1st node explants. Τhe high production of microshoots from basal single node explants in subcultures strengthens our hypothesis that axillary buds of wild plants are gradually deactivated due to age-induced senescence and/or to exposure to stress conditions (e.g., gradual reduction of water supply) which occur from late spring and during summer in the natural environment of plants.

Microshoot length was favoured at 25 °C compared to 15 °C, regardless node position, a finding which is similar with other *Origanum* species [26]. The rapid elongation of the shoot apex has also been observed in *O. vulgare* in vitro culture at 25 ± 1 °C by Oana et al. [21]. Moreover, microplants produced by shoot apex formed longer shoots compared to those produced by the 1st and 5th node. It could be suggested that the shorter microshoots produced by axillary nodes may reflect the higher competition in those single node explants which formed more microshoots than shot apex. The propagules (microshoots) produced in this stage (Stage II) can also be used for further cycles of multiplication. In such cases, short length of microshoots does not favour cutting to obtain new single node explants for subsequent subcultures [48]. Techniques such as the subculture under high temperatures (e.g., 25 °C in this study) or the addition of gibberellic acid in the medium can lead to longer microshoots and facilitate more cycles of multiplication (Alexopoulos unpublished data), which is essential for the commercial micropropagation of the species.

The number of leaves per microplant was not significantly affected by the node position in the case of 15 °C, whereas at 25 °C the highest number of leaves was recorded for explants collected from the 1st node without being significantly different for the apical shoot explant (Table 4). In any case, it could be suggested that the number of leaves produced in vitro is adequate for the photosynthetic activity of the plantlets during the early stages of ex vitro cultivation, as it was reported in other *Origanum* species, e.g., *O. vulgare* [53], and *O. syriacum* [54].

All single node explants collected from the shoot tip produced rooted microplants at both tested temperatures, being significantly different from 5th node explants at 15 °C and both axillary explants at 25 °C (Table 4). This finding is in agreement with results reported by Ma et al. [55] in rose species. Moreover, the percentage of rooted microplants at 35 days after the start of the subcultures was higher in microplants produced from single node explants of the 1st and 5th node at 15 °C in comparison with those at 25 °C. In contrast, no significant differences were recorded between the tested temperatures at 50 days after the start of the subcultures. In the case of *Camellia sinensis* [(L.) Kuntze] temperature affected microplant rooting in vitro [56], with temperatures lower than 15 °C or higher than 35 °C resulting in reduced percentage of rooting. In general, constant temperatures during proliferation and rooting, as those tested in our study, have been reported to be more favorable than alternating day and night temperatures [57].

Despite the slow rate of root formation in *O. scabrum* microplants cultured in medium without the addition of auxins, the percentage of rooted microplants is as high as in other *Origanum* species, e.g., *O. vulgare* cultured in media containing IBA or NAA [53,58]. The production of rooted microshoots in media without auxins has also been observed in other Mediterannean Lamiaceae species, such as *Thymus satureioides* (Coss.) [59], *Lavandula officinalis* (Chaix) [60], *Teucrium capitatum* (L.) [61], *Clinopodium* (Mill.) (syn. *Calamintha* L.) spp. [62,63], *Salvia* spp. [64], *Anthyllis hermanniae* (L.) [65], *O. vulgare* ssp. *hirtum* [24], and *O. ehrenbergii* and *O. syriacum* [13].

### 2.3. Acclimatization and Ex Vitro Survival of Plantlets

**Figure 3:**
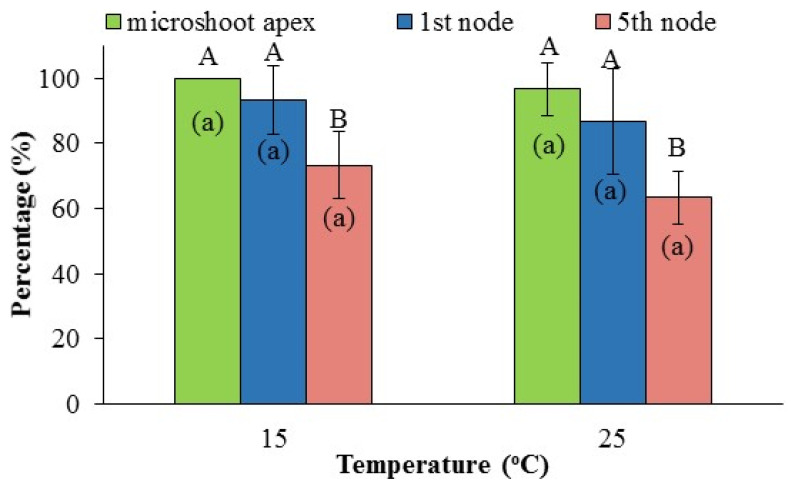
The effect of temperature during in vitro culture and node position on mother microshoot on mean percentage of plantlet ex vitro survival after four months. Different capital letters indicate statistically significant difference between the node positions in each temperature level separately, according to the LSD test (*p* ≤ 0.05). Different lower-case letters indicate statistically significant difference between the temperatures in each node position separately, according to the *t*-test (*p* ≤ 0.05).

The percentage (%) of plantlet survival grown ex vitro in greenhouse conditions for 4 months was not affected by the temperature during proliferation and rooting in the subcultures. However, the survival rate was affected by the position of the collection point of explants (Figure 3). In particular, plantlets produced from shoot apex and 1st node explants recorded higher survival rate than those obtained from the 5th node explants (93.3–100%, 86.7–93.3% and 66.7–73.3%, respectively). This observation could be associated with the lower percentage of rooted microplants produced from the 5th node explants (Table 4), indicating difficulties in root production and lower acclimatization rates of the plantlets [46]. In general, the survival rates recorded in our study are considered high enough and they are similar to those reported for *O. vulgare* × *applii* [14], *O. glandulosum* (Desf.) [25] and *O. sipyleum* [66] and suitable for the successful transplantation of plantlets and the establishment of new crops in the open field. Moreover, the high survival rates of plantlets recorded in our study confirms the success of the suggested micropropagation protocol [37] without requiring special treatments for hardening and acclimatization [67].

## 3. Materials and Methods

### 3.1. Plant Material

**Figure 4:**
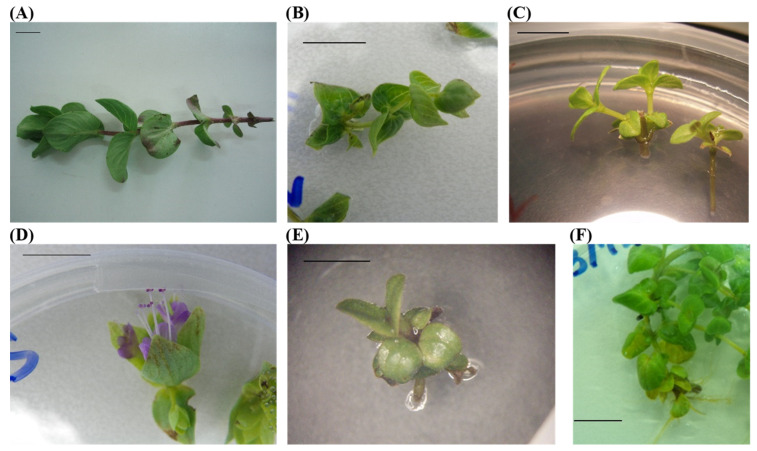
(**A**) Wild plant stem collected on April 20 and used for cutting nodes (explants). (**B**) Microshoot produced by in vitro culture of the shoot apex of a wild plant (Stage I). (**C**) Microshoots produced by in vitro culture of an axillary node of a wild plant (Stage I). (**D**) Inflorescence development during the in vitro culture of a shoot apex collected on 20 June (Stage I). (**E**) Early growth of a microplant produced at 15 °C using a microplant-shoot apex as explant (Stage ΙΙ). (**F**) Rooted microplant produced at 25 °C using the first lateral node from the top of the microplant as explant (Stage ΙΙ). Horizontal bars in each photo depict the length of 1 cm.

*O. scabrum* stems were collected from wild plants in the broad area of Dyrrachion of the Arcadia Prefecture in Peloponnese, Greece (altitude 900–950 m above the see level, 37°10′50″ N, 22°12′20″ E, WG S84). Stems (Figure 4A) were cut from the mother plants at the soil surface, without removing the underground part-rhizome and roots. Then, stems were placed in a damp cloth (to keep them fresh and moistened) and transferred to the Laboratory of Agronomy at the University of the Peloponnese for the establishment of in vitro cultures, as described in Section 3.2 below.

### 3.2. Establishment of In Vitro Cultures (1st Experiment: Stage I)

For the establishment of in vitro culture, the effect of collection date of explants and the effect of node position on the stem was studied. Stems from wild plants were collected on five consecutive dates, namely 20 April, 20 May, 20 June, 20 July and 20 August. Shoots were thoroughly rinsed with running tap water for 10 min and then cut into 5–7 mm long sections, each containing a single node or only the shoot apex (single node culture). Separate explants from (a) the shoot apex, (b) the 1st node, (c) the 3rd node below the shoot apex, and (d) the 5th node below the shoot apex were used for the establishment of in vitro cultures.

Surface sterilization of explants was performed in an airflow cabinet (CDR LMR-185/75, Athens, Greece). The explants were immersed for 10 min in a sodium hypochlorite solution (0.5% *w*/*v*), where Tween 20 (Sigma, Roedermark, Germany) was added (three drops per 100 mL solution). During surface sterilization, explants were shaken manually without injuring the explants in order to increase the effectiveness of the sterilization method. The explants were rinsed with sterile water three times, for three minutes each time.

Εxplants, in groups of five, were transferred under aseptic conditions to Petri dishes (diameter 9 cm) filled with 33 mL of semi-solid nutrient medium MS (Murashige and Skoog M5519 basal mixture, Sigma-Aldrich, St. Louis, MO, USA) modified with agar (Serva, Heidelberg, Germany) and sucrose (Serva, Heidelberg, Germany) at concentrations of 4 g L^−1^ and 30 g L^−1^, respectively. The pH of the medium was adjusted to 5.8. Petri dishes were placed in an incubation chamber with 16 h light/8 h dark at 18 °C and 12 °C, respectively, and under fluorescent light (33 μMol m^−2^ s^−1^).

For each explant collection date and each node position οn the stem, 60 explants were used. The explants remained in the incubation chamber for 45 days, and the percentage (%) of responding explants was measured 25 days after establishment the in vitro culture, while the number of shoots and the number of new nodes per microplant were also measured 45 days after the establishment of the in vitro culture.

### 3.3. Production of Suitable Microplants (2nd Experiment: Stage II)

Shoot proliferation and the production of suitable microplants was achieved in subcultures of single node explants from microplants produced in Stage I. For this purpose, unbranched microplants obtained from 1st-node explants collected on May 20 remained for additional 30 days in culture for further growth (75 days in total from the establishment of the in vitro cultures). After that time period, 60 microplants with microshoots with at least 6 nodes (mean number of nodes per micropalnt = 7.1) were selected for receiving single node explants. During the subculture of the single node explants, the effect of node position on the microshoot and the effect of temperature were also studied. Single node explants from different positions of the microshoots were taken separately, i.e., the apex of the microshoot, and the 1st and 5th node below the microshoot apex. The single node explants were transferred, in groups of five under aseptic conditions in Petri dishes (diameter 9 cm; a total of 12 Petri dishes with five single node pieces for each different position; 180 single nodes in total), following the protocol described in Stage I. Half of the Petri dishes were transferred to a growth chamber at 15 °C (i.e., 30 single node explants for each different position; 90 single node explants in total) and the rest were put at 25 °C (i.e., 30 single node explants for each different position; 90 single node explants in total). The light conditions were similar to Stage I.

The number of microshoots and the number of leaves per microplant, as well as the length of the microshoots and the percentage of rooted microplants were measured at 35 and 50 days after the start of the subcultures.

### 3.4. Acclimatization and Ex Vitro Survival of Plantlets

**Figure 5:**
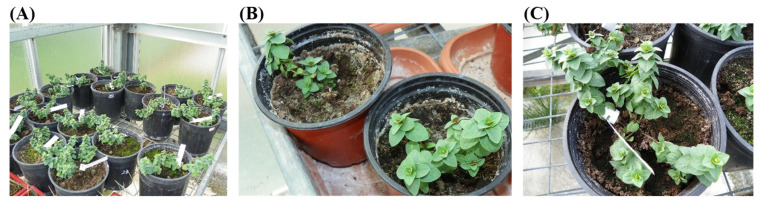
(**A**) Ex vitro growth of microplants in an unheated greenhouse 4 months after transplantation, (**B**) Ex vitro growth of microplants in 1 L pots (60 days after the 1st transplantation), (**C**) Ex vitro growth of microplants in 2 L pots (120 days after the 2nd transplantation).

Fifty days after the start of the subcultures (Stage ΙΙ), 15 rooted microplants from each treatment (temperature x node position) were transferred to 1 L pots filled with peat (KTS2, Klasmann-Deilmann Gmbh, Geeste, Germany) that contained 280–360 mg L^−1^ N, 320–410 mg L^−1^ P_2_O_5_ and 370–460 mg L^−1^ K_2_O (1:1; *v*/*v*). Before transplantation, microplants’ roots were thoroughly washed with deionized water to remove the remains of the culture medium. The pots were placed in a “walk in” chamber with controlled conditions, e.g., constant temperature at 20 °C (16 h light/8 h dark), light intensity of 45 μMol m^−2^ s^−1^ with the use of fluorescent lamps, and average relative humidity of 80% (Figure 5A). At this stage, plantlets were covered with transparent plastic. After 15 days, plantlets were transferred to an unheated greenhouse where they remained for 45 days (Figure 5B). Then, the plantlets were transplanted into 2 L pots (Figure 5C) with the same peat substrate and remained there for further growth (temperature ranged between 11.2 and 30.8 °C). During that period, fertilization was carried out every 15 days with 50 mL aqueous solution, using 20-20-20 (N-P-K) plus trace elements (Nutrileaf, Miller, PA, USA) fertilizer at a concentration of 1 g per 10 L. After 2 months, the ex vitro survival rate (%) of plants was measured (4 months, in total, after plants transfer from the in vitro cultures).

### 3.5. Statistical Analysis

Stage I and Stage II experiments were two-factor factorial (Stage I: factor A—explant collection date, factor B—node position; Stage II: factor A—temperature, B—node position) laid out according to the completely randomized and the split-plot design, respectively. In both experiments, the two-way ANOVA showed a significant interaction between the tested factors. Therefore, the effect of each factor was estimated separately at each level of the other factor. Significance of differences between treatment was assessed by the least significant difference (LSD) test at *p* ≤ 0.05. In Stage II experiment, comparisons of the two temperatures at each node position were made using Student’s *t*-test at *p* ≤ 0.05.

## 4. Conclusions

In this work, in vitro propagation parameters of *Origanum scabrum* were studied, focusing on the collection date of explants and node position on mother plant stem at the establishment stage of in vitro cultures (Stage I), as well as on the temperature and node position on mother microshoots in the subculture explants (Stage II). The results suggest that the optimum time to collect explants from wild plants (preferably the 1st visible node below the shoot tip) should be between April and May (during the vegetative stage of the plant). Moreover, single node explants containing one bud (i.e., shoot tip and axillary nodes) should be taken from microshoots produced in vitro and transferred in media without plant growth regulators for subculture (proliferation and rooting) at temperatures of 15–25 °C. These conditions allow high percentages of rooted plants and high ex vitro survival rates (66.7–100%). In conclusion, the present work describes an efficient and low-cost protocol for the propagation of *O. scabrum* aiming to facilitate the large scale production of propagating material, which is prerequisite for the establishment and commercial exploitation of the species in the pharmaceutical and horticultural industries, and the for the preservation of genetic plant material considering the in situ and ex situ conservation of this rare plant species.

## Figures and Tables

**Table 1:** The effect of explant collection date and node position on the mother plant shoot on mean percentage (%; mean ± SD) of explant response 25 days after the establishment of in vitro cultures.

	Shoot Apex	1st Node	3rd Node	5th Node
20 April	93.3 A(a) ± 10.328	90.0 A(a) ± 10.954	56.7 A(b) ± 8.165	23.3 A(c) ± 8.165
20 May	90.0 A(a) ± 10.954	96.7 A(a) ± 8.165	23.3 B(b) ± 15.055	6.7 B(c) ± 10.338
20 June	80.0 A(a) ± 12.649	76.7 B(a) ± 8.165	6.7 C(b) ± 10.328	3.3 B(b) ± 8.165
20 July	53.3 B(a) ± 13.330	26.7 C(b) ± 10.328	3.3 C(c) ± 8.165	3.3 B(c) ± 8.165
20 August	16.7 C (a) ± 15.055	3.3 D(b) ± 8.165	3.3 C(b) ± 8.165	0.0 B(b) ± 0.000

Means within each column followed by the same capital letter are not significantly different at *p* ≤ 0.05. Means within each row followed by the same lowercase letter (in parenthesis) are not significantly different at *p* ≤ 0.05.

**Table 2:** The effect of explant collection date and node position on the mother plant on mean number of microshoots per microplant (mean ± SD) 45 days after the establishment of in vitro cultures.

	Shoot Apex	1st Node	3rd Node	5th Node
20 April	1.00 A(b) ± 0.0000	1.55 AB(a) ± 0.0548	0.70 A(c) ± 0.2678	0.50 A(c) ± 0.8367
20 May	1.00 A(b) ± 0.0000	1.58 A(a) ± 0.0229	0.60 A(c) ± 0.4183	0.50 A(c) ± 0.7071
20 June	1.00 A(b) ± 0.0000	1.25 B(a) ± 0.2739	0.50 A(c) ± 0.7071	0.00 B(d) ± 0.0000
20 July	1.00 A(a) ± 0.0000	0.67 C(b) ± 0.4082	0.00 B(c) ± 0.0000	0.00 B(c) ± 0.0000
20 August	0.50 B(a) ± 0.5774	0.00 D(b) ± 0.0000	0.00 B(b) ± 0.0000	- ^†^

Means within each column followed by the same capital letter are not significantly different at *p* ≤ 0.05. Means within each row followed by the same lowercase letter (in parenthesis) are not significantly different at *p* ≤ 0.05. ^†^ no response.

**Table 3:** The effect of explant collection date and node position on the mother plant shoot on mean number of nodes per microplant (mean ± SD) 45 days after the establishment of the in vitro cultures.

	Shoot Apex	1st Node	3rd Node	5th Node
20 April	2.08 BC(a) ± 0.2041	2.60 A(a) ± 0.8556	0.97 A(b) ± 0.5833	0.25 A(c) ± 0.4183
20 May	2.33 AB(a) ± 0.5164	2.60 A(a) ± 0.8295	0.90 A(b) ± 0.5477	0.50 A(b) ± 0.7071
20 June	2.67 A(a) ± 0.5164	1.60 B(b) ± 0.3272	0.50 B(c) ± 0.7071	^††^
20 July	1.58 C(a) ± 0.4916	1.17 B(b) ± 0.2582	^††^	^††^
20 August	0.50 D ± 0.5774	^††^	^††^	- ^†^

Means within each column followed by the same capital letter are not significantly different at *p* ≤ 0.05. Means within each row followed by the same lowercase letter (in parenthesis) are not significantly different at *p* ≤ 0.05. ^†^ no viable explant; ^††^ no microplants were produced (see Table 2).

**Table 4:** The effect of temperature during culture and node position on the mother microshoot on mean number of shoots, leaves and roots per microplant and mean microshoot length after 35 and 50 days of subculture start (mean ± SD).

	35 Days			50 Days		
	Shoot Apex	1st Node	5th Node	Shoot Apex	1st Node	5th Node
Number of microshoots per microplant						
15 °C	1.0 A(b) ± 0.000	2.0 A(a) ± 0.000	1.8 A(a) ±0.346	1.0 A(b) ± 0.000	2.0 A(a) ± 0.000	1.8 A(a) ± 0.231
25 °C	1.0 A(b) ± 0.000	2.0 A(a) ± 0.000	1.0 B(b) ±0.173	1.0 A(b) ± 0.000	2.0 A(a) ± 0.00	1.6 A(a) ± 0.321
Microshoot length (cm)						
15 °C	0.97 B(a) ± 0.156	0.67 B(b) ± 0.114	0.28 B(c) ± 0.056	1.61 B(a) ± 0.335	0.71 B(b) ± 0.180	0.43 Β(b) ± 0.157
25 °C	3.53 A(a) ± 0.555	2.00 A(b) ± 0.363	0.57 A(c) ± 0.128	3.90 A(a) ± 0.627	2.25 A(b) ± 0.304	0.93 A(c) ± 0.189
Number of leaves per microplant						
15 °C	11.0 A(a) ± 2.193	11.0 B(a) ± 1.201	10.6 A(a) ± 1.803	14.0 A(a) ± 2.155	14.0 B(a) ± 1.604	13.7 A(a) ± 2.458
25 °C	13.5 A(ab) ± 3.012	17.5 A(a) ± 3.089	9.3 A(b) ± 2.120	16.8 A(ab) ± 2.951	20.0 A(a) ± 3.272	11.3 A(b) ± 2.330
Percentage (%) of rooted microplants						
15 °C	100 A(a) ± 0.000	90.0 A(a) ± 10.000	76.7 A(b) ± 5.774	100 A(a) ± 0.000	93.3 A(a) ± 5.774	76.7 A(b) ± 5.774
25 °C	100 A(a) ± 0.000	46.7 B(b) ± 11.547	40.0 B(b) ± 0.000	100 A(a) ± 0.000	80.0 A(b) ± 17.321	63.3 A(c) ± 15.275

Means within each column followed by the same capital letter are not significantly different at *p* ≤ 0.05. Means within each row (separately for each day of measurement) followed by the same lowercase letter (in parenthesis) are not significantly different at *p* ≤ 0.05.

## Data Availability

Data are available upon request.
